# Impact of New-Onset Conduction Disturbances following Transcatheter Aortic Valve Replacement on Outcomes: A Single-Center Study

**DOI:** 10.1155/2023/5390338

**Published:** 2023-05-31

**Authors:** Claudia See, Yanting Wang, Haocheng Huang, Helen Parise, Yiping Yang, Daniela Tirziu, Dominic P. Francese, Nikolaos Papoutsidakis, Eric Bader, Ryan K. Kaple, Michael Cleman, Alexandra J. Lansky, John K. Forrest

**Affiliations:** ^1^From the Section of Cardiovascular Medicine, Yale School of Medicine, New Haven, CT, USA; ^2^Hackensack Meridian Jersey Shore University Medical Center, NJ 07753, Neptune Township, USA; ^3^Cardiovascular Medicine Clinical Research Analytics Group, Yale School of Medicine, New Haven, CT, USA; ^4^Barts Heart Centre, London and Queen Mary University of London, London, UK

## Abstract

**Background:**

Transcatheter aortic valve replacement (TAVR) is known to increase the incidence of conduction disturbances compared to surgical aortic valve replacement; however, there are limited data on the impact and duration of these conduction disturbances on longer term outcomes.

**Objective:**

To determine the differential impact of persistent versus nonpersistent new-onset conduction disturbances on TAVR-related complications and outcomes.

**Methods:**

This is a single-center retrospective analysis of 927 consecutive patients with aortic stenosis who underwent TAVR at Yale New Haven Hospital from July 2012 to August 2019. Patients with new-onset conduction disturbances within 7 days following TAVR were selected for this study. Persistent and nonpersistent disturbances were, respectively, defined as persisting or not persisting on all patient ECGs for up to 1.5 years after TAVR or until death.

**Results:**

Within 7 days after TAVR, conduction disturbances occurred in 42.3% (392/927) of the patients. Conduction disturbances persisted in 150 (38%) patients and did not persist in 187 (48%) patients, and 55 (14%) patients were excluded for having mixed (both persistent and nonpersistent) disturbances. Compared with nonpersistent disturbances, patients with persistent disturbances were more likely to receive a PPM within 7 days after the TAVR procedure (46.0% versus 4.3%, *p* < 0.001) and had a greater unadjusted 1-year cardiac-related and all-cause mortality risk (HR 2.54, *p*=0.044 and HR 1.90, *p*=0.046, respectively).

**Conclusion:**

Persistent conduction disturbances were associated with a greater cardiac and all-cause mortality rate at one year following TAVR. Future research should investigate periprocedural factors to reduce persistent conduction disturbances and outcomes beyond one year follow-up.

## 1. Introduction

Transcatheter aortic valve replacement (TAVR) is approved as an alternative to surgical aortic valve replacement across the spectrum of surgical risk [1, 2]. Despite advances in valve technology and implantation techniques, conduction disturbances are a known complication of TAVR related to clinical and procedural factors, including implantation depth, valve oversizing, and valve type [3]. Conduction disturbances are reported to occur in 31–45% of the patients depending on the type of valve implanted [4–6], with new-onset left bundle branch block (LBBB) reported in 7–65% [7, 8] and new-onset atrial fibrillation in 5–13% of patients [1, 2, 9–11], and resulting in permanent pacemaker (PPM) implantation in cases of high degree atrioventricular block (AVB) [12, 13]. Prior studies have shown that persistent new-onset conduction disturbances after TAVR are associated with worse outcomes, including increased risk of cardiac and all-cause mortality with new-onset LBBB [13, 14] and increased risk of all-cause mortality with new-onset atrial fibrillation [15]. However, the differential prognostic impact of persistent versus nonpersistent new-onset disturbances on TAVR-related complications and outcomes is not well understood. We address this question in a single-center TAVR registry.

## 2. Methods

**Figure 1:**
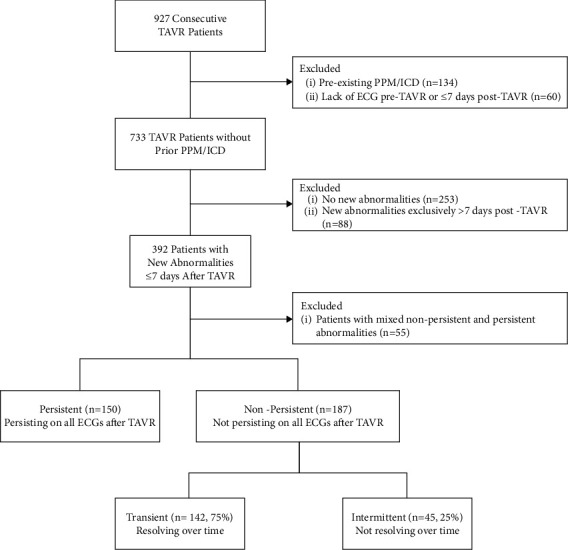
Patient flow chart. Breakdown of patients into groups by conduction disturbance. ECG = electrocardiogram; ICD = implantable cardioverter defibrillator; PPM = permanent pacemaker; TAVR = transcatheter aortic valve replacement.

This is a single-center, retrospective analysis of consecutive patients with severe symptomatic aortic stenosis who underwent TAVR at Yale New Haven Hospital from July 2012 to August 2019. We include only patients who had new-onset conduction disturbances within 7 days after TAVR. Patients were excluded if they had a pre-existing PPM and/or implantable cardioverter defibrillator (ICD) or if electrocardiogram (ECG) was not performed or available before or within 7 days after TAVR. Patients with new-onset disturbances after 7 days after TAVR with no new-onset disturbances or with mixed (both persistent and nonpersistent) disturbances were also excluded (Figure 1).

For all patients, ECGs were performed before, immediately after, and up to 1.5 years after TAVR. Conduction defect is defined on ECG as left or right bundle branch block, intraventricular conduction delay, 2 : 1, 3 : 1, and 4 : 1 block, bifascicular block, left anterior and posterior fascicular block, first-degree atrioventricular block, second degree atrioventricular block Mobitz Type I and II, third-degree atrioventricular block, and atrial fibrillation or flutter. New-onset persistent disturbances were defined as new disturbances that persisted on all ECGs for up to 1.5 years after TAVR or until death. New-onset nonpersistent disturbances were defined as disturbances that did not persist on all ECGs for up to 1.5 years after TAVR or until death. All ECG readings were conducted by independent board-certified cardiologists and entered into the electronic medical record (EMR). Chart review was conducted by querying the EMR database. The findings were classified based on the American College of Cardiology/American Heart Association/Heart Rhythm Society recommendations [16]. PPM placement was clinically based by the TAVR operator in consultation with an electrophysiologist.

Data acquisition, monitoring, and outcome assessments were performed according to the STS/ACC Transcatheter Valve Therapy (TVT) Registry [17, 18]. Eligibility for TAVR was based on decision of the multidisciplinary heart team consisting of experienced surgeons, interventional cardiologists, and imaging specialists. This study was approved by the Yale Institutional Review Board (No. 2000028604). Device success was defined as successful vascular access, delivery, and deployment of a single device in the proper anatomic location, appropriate performance of the prosthetic heart valve (aortic valve area >1.2 cm^2^ and mean aortic valve gradient <20 mm Hg or peak velocity <3 m/s without moderate or severe prosthetic valve aortic regurgitation) and the successful retrieval of the delivery system. Implant success was defined as the correct positioning of a single device in the proper anatomic location.

The primary endpoint was all-cause mortality at 1 year after TAVR. Secondary endpoints included the major adverse cardiac events (MACEs) at 30 days, defined as a composite of death, myocardial infarction (MI), stroke, valve-related hospitalization, and cardiac arrest, according to the Valve Academic Research Consortium-2 (VARC-2) definitions [19]. Other adverse events at 30 days included major bleeding, major vascular complications, and all-cause mortality. Major vascular complication was defined as a composite of the major vascular access site complication or unplanned vascular surgery, annular rupture, aortic dissection, or perforation with or without tamponade. All 30-day adverse events were adjudicated by board-certified cardiologists using a combination of site-reported clinical information and targeted chart reviews. Mortality and cause of death at 1 year were determined by the chart review of electronic medical records, publicly accessible online obituaries, and the Centers for Disease Control and Prevention National Death Index registry.

### 2.1. Statistical Analysis

Descriptive statistics for continuous variables included mean, standard deviation, and sample size for each treatment group. Categorical variables were summarized using frequencies, percentages, and sample size for each treatment group. Categorical variables were compared using the Pearson *χ*^2^ test or the Fisher exact test. Continuous variables were compared with Student's *t*-test or the Wilcoxon rank-sum test if the data failed to meet the assumption for normality per the Shapiro–Wilk test. For time-to-event data, Kaplan–Meier estimates were calculated and displayed graphically. The univariable Cox proportional hazard regression model presented hazard ratios (HRs) with 95% confidence intervals. All analyses were conducted using SAS version 9.4 (SAS Institute). No imputation was considered for missing values. Values of *p* < 0.05 were considered statistically significant.

## 3. Results

### 3.1. Baseline Characteristics

A total of 392 patients developed at least 1 new-onset conduction disturbance within 7 days after TAVR. Of the 392, conduction disturbances were persistent in 150 (38%) patients, nonpersistent in 187 (48%) patients, and mixed in 55 (14.0%) patients (Figure 1). Patients with mixed disturbances were excluded from analysis. Of the 187 patients with nonpersistent disturbances, 142 (75%) resolved over time while 45 (25%) did not (Figure 1).

Baseline and procedural TAVR characteristics are shown in Table 1. At the baseline, the patients with persistent new-onset disturbances had more prior conduction disturbances (48.0% versus 26.2%, *p* < 0.001) and lower LVEF (56.0 ± 12.5 versus 61.4 ± 11.8, *p* < 0.001) compared with the patients with nonpersistent new-onset disturbances. Additionally, the patients with persistent new-onset disturbances had longer 5-meter walk times (8.60 seconds versus 7.96 seconds, *p*=0.03). The STS risk score was comparable between the persistent and nonpersistent groups (6.53% versus 6.30%, *p*=0.62).

### 3.2. Conduction Disturbances

The most common new-onset conduction disturbances found within 7 days after TAVR implantation were LBBB (23%), intraventricular conduction delay (IVCD) (14%), first-degree AVB (13%), and third-degree AVB (12%) (Supplemental Table 1).

### 3.3. TAVR Procedural Characteristics and Outcomes

Procedural TAVR characteristics of the study population are summarized in Table 1. Most cases were elective (89.9%) and performed via transfemoral access (89.9%) under moderate sedation (61.9%) or general anesthesia (36.9%). Overall device success was 97.9% and implantation success was 98.2%, with no differences in the two groups. The use of antiplatelet and antithrombotic medications at discharge was similar between the two groups.

The 337 patients received a balloon-expandable (50.1%) or self-expandable (49.9%) valve (Table 1). All balloon-expandable systems were SAPIEN, SAPIEN XT, or SAPIEN 3 (Edwards Lifesciences), and all self-expandable valves were CoreValve, Evolut R, or Evolut Pro valve systems (Medtronic). The incidence of new-onset persistent conduction disturbances was similar in patients receiving a balloon-expandable (52.7%) and self-expanding (47.3%) valves (Table 1). A breakdown of conduction disturbances by the valve type is provided in Supplemental Table 2.

### 3.4. Outcomes at 30 Days

The occurrence of MACE at 30 days post procedure was low and similar in both groups (Table 2). There were no significant differences between the persistent and nonpersistent groups for 30-day unadjusted or adjusted rates of MACE and mortality (Table 3).

### 3.5. Outcomes at 1 Year

**Figure 2:**
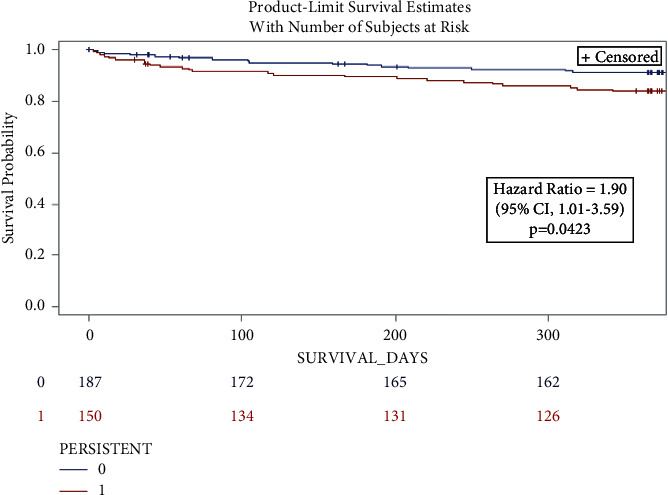
Kaplan–Meier curves for survival at 1 year for patients with persistent versus nonpersistent conduction disturbances. Patients with persistent conduction disturbances have a nonsignificant trend towards greater 1-year mortality compared with nonpersistent patients. CI = confidence interval.

Compared with the patients with nonpersistent disturbances, patients with persistent disturbances had a greater risk of cardiac-related and all-cause mortality in unadjusted analysis (HR 2.54, 95% 1.02–6.29 and HR 1.90, and 95% 1.01–3.59, respectively) (Figure 2).

### 3.6. PPM Implantation

**Figure 3:**
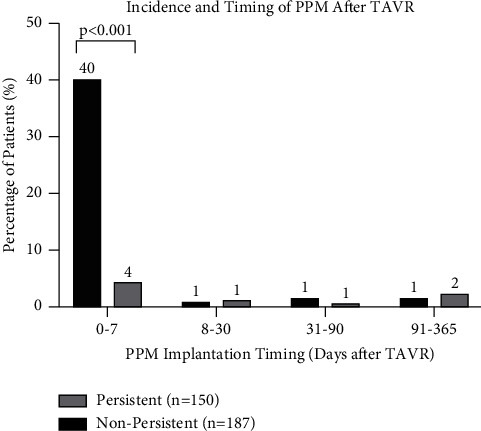
Incidence and timing of permanent pacemaker or implantable cardioverter defibrillator after TAVR for patients with persistent versus nonpersistent conduction disturbances. Note: pairwise analyses for 8–365 day groups not conducted because of cell count <5. ICD = implantable cardioverter defibrillator; PPM = permanent pacemaker; TAVR = transcatheter aortic valve replacement.

Patients with persistent disturbances were more likely to receive a PPM within 7 days after the TAVR procedure compared with patients with nonpersistent disturbances (46.0% versus 4.3%, *p* < 0.001) (Figure 3). Patients with persistent disturbances had greater 30-day PPM rates after TAVR compared with the patients with nonpersistent disturbances (46.7% versus 5.9%, *p* < 0.001). In the persistent group, the indications for 30-day PPM placement were complete AVB, bundle branch block, and primary prevention in 90%, 8%, and 2% of the patients, respectively. In the nonpersistent group, the indications for 30-day PPM were complete AVB, high-grade AVB, and bundle branch block in 70%, 20%, and 10% of the patients, respectively. One-year PPM rates according to the valve type were 12.8% for balloon-expandable valves and 11.0% for self-expanding valves.

## 4. Discussion

This study compared characteristics and outcomes for patients undergoing TAVR who had persistent versus nonpersistent new-onset conduction disturbances postprocedure. The main findings of our study are as follows: (1) 38% of the patients with new-onset conduction disturbances after TAVR had persistent disturbances and 48% had nonpersistent disturbances, (2) persistent disturbance patients were more likely to receive a PPM within 7 days after the TAVR procedure, and (3) persistent disturbance patients had a greater unadjusted 1-year mortality risk.

Prior studies have shown that persistent new-onset conduction disturbances after TAVR are associated with worse outcomes, including the increased risk of cardiac mortality and PPM implantation at 1-year follow-up for new-onset LBBB [14], increased all-cause and cardiovascular mortality at 2-year follow-up for new-onset persistent LBBB [13], and increased mortality at a median follow-up of 305 days for new-onset atrial fibrillation [15]. However, this is the first study to compare the differential impact of persistence on outcomes.

In our study, 38% of the patients with new-onset disturbances had persistent only disturbances, which is similar to prior smaller scale reports with the SAPIEN valve [20]. Persistent conduction disturbances were associated with an increased risk for 1-year cardiac-related and all-cause mortality based on univariable analysis (Table 2; Figure 2 Kaplan–Meier). Additionally, persistent disturbance patients had signs of worse baseline health; greater 5-meter walk score, more prior conduction disturbances, and lower baseline LVEF (Table 1).

Our study includes newer generation TAVR systems (Edward Sapien 3 valve and Medtronic Evolut R and Pro valves), which should have improved valve design and implantation techniques, lower pacemaker rates [21], and better outcomes [22–25]. Our study also includes intermediate STS risk patients, an indication expanded in 2016 after the PARTNER-2 SURTAVI trial [26]. The results of our study suggest that the persistence of disturbances may outweigh these beneficial changes and contribute to increased mortality. Additionally, the similar STS mortality risk scores between persistent and nonpersistent disturbance patients (6.53% vs. 6.30%, respectively, *p* = 0.62) suggest that the persistent nature of the disturbances may play a bigger role in mortality than expected.

The persistence of conduction disturbances may be explained by modifiable causes such as mechanical trauma from the implantation depth, heart valve oversizing, and radial force of the heart valve at the left ventricular outflow tract (LVOT) level or nonmodifiable causes such as LVOT geometry, anatomical variability of the conduction system, and distribution and amount of calcification [27]. Further research is needed to assess and educate operators on the factors contributing to persistent conduction disturbances.

## 5. Limitations

Our study represents a real-world population of patients undergoing TAVR at a single-center tertiary referral center. As a retrospective study, it has inherent limitations and is subject to bias, and being conducted at a single center, the results may not be generalizable. Nonpersistent conduction disturbances that arose immediately after TAVR but resolved before the postprocedure ECG were not captured, and there are likely other unmeasured confounders affecting 30-day and 1-year survival. Comparison of individual conduction disturbances and multivariable regression analysis could not be conducted because the respective numbers of patients and outcome events were too low. Longer follow-up past 1 year or a larger patient population may be needed to improve statistical power. Lastly, we did not examine differences in outcomes according to newer versus older generation TAVRs.

## 6. Conclusion

This single-center study suggests that persistent conduction disturbances after TAVR lead to worse 1-year outcomes when compared with nonpersistent disturbances. Additionally, these results suggest that patients with new-onset persistence disturbances within seven days after TAVR have greater PPM/ICD rates when compared with patients with nonpersistent new disturbances after TAVR. Future research should look at periprocedural factors to reduce persistent conduction disturbances in TAVR patients.

## Figures and Tables

**Table 1:** Baseline and procedural characteristics.

	Overall	Persistent	Nonpersistent	*p* values
	*N* = 337	*N* = 150	*N* = 187	
Age (year)	81.7 ± 7.8 (337)	81.8 ± 8.4 (150)	81.6 ± 7.3 (187)	0.55
Male (sex)	178/337 (52.8%)	88/150 (58.7%)	90/187 (48.1%)	0.054
Body mass index (kg/m^2^)	29.28 ± 6.71 (336)	29.85 ± 7.35 (150)	28.82 ± 6.14 (186)	0.34
Hypertension	298/337 (88.4%)	129/150 (86.0%)	169/187 (90.4%)	0.21
Diabetes	117/337 (34.7%)	57/150 (38.0%)	60/187 (32.1%)	0.26
Chronic obstructive pulmonary disease	77/336 (22.9%)	29/149 (19.5%)	48/187 (25.7%)	0.48
Prior myocardial infarction	78/335 (23.3%)	35/148 (23.6%)	43/187 (23.0%)	0.89
Atrial fibrillation/flutter	121/335 (36.1%)	57/149 (38.3%)	64/186 (34.4%)	0.47
Prior conduction disturbance	121/335 (36.1%)	72/150 (48.0%)	49/185 (26.5%)	<0.001
NYHA functional class III/IV	282/336 (83.9%)	129/150 (86.0%)	153/186 (82.3%)	0.35
Carotid stenosis	46/332 (13.9%)	13/148 (8.8%)	33/184 (17.9%)	0.02
Unilateral	24/332 (7.2%)	8/148 (5.4%)	16/184 (8.7%)	0.25
Bilateral	22/332 (6.6%)	5/148 (3.4%)	17/184 (9.2%)	0.03
Prior stroke	24/337 (7.1%)	8/150 (5.3%)	16/187 (8.6%)	0.25
Prior percutaneous coronary intervention	110/337 (32.6%)	57/150 (38.0%)	53/187 (28.3%)	0.06
Prior coronary artery bypass grafting	57/336 (17.0%)	26/150 (17.3%)	31/186 (16.7%)	0.87
Prior transient ischemic attack	20/336 (6.0%)	8/150 (5.3%)	12/186 (6.5%)	0.67
Peripheral arterial disease	86/337 (25.5%)	42/150 (28.0%)	44/187 (23.5%)	0.35
Left ventricular ejection fraction (%)	59.0 ± 12.4 (333)	56.0 ± 12.5 (147)	61.4 ± 11.8 (186)	<0.001
<30%	9/333 (2.7%)	4/147 (2.7%)	5/186 (2.7%)	1.00
30–45%	38/333 (11.4%)	25/147 (17.0%)	13/186 (7.0%)	0.004
45–55%	33/333 (9.9%)	21/147 (14.3%)	12/186 (6.5%)	0.02
≥55%	253/333 (76.0%)	97/147 (66.0%)	156/186 (83.9%)	<0.001
Glomerular filtration rate (mL/min)	61.1 ± 36.4 (333)	62.0 ± 45.5 (148)	60.3 ± 27.2 (185)	0.39
STS score PROM (%)	6.40 ± 3.87 (305)	6.53 ± 3.90 (135)	6.30 ± 3.86 (170)	0.62
5-meter walk test, sec	8.23 ± 2.58 (251)	8.60 ± 2.70 (108)	7.96 ± 2.46 (143)	0.03

*Device type*				
Self-expanding	169/337 (50.1%)	71/150 (47.3%)	98/187 (52.4%)	0.36
Corevalve	38/337 (11.3%)	17/150 (11.3%)	21/187 (11.2%)	0.98
Evolut pro	74/337 (22.0%)	30/150 (20.0%)	44/187 (23.5%)	0.44
Evolut R	57/337 (16.9%)	24/150 (16.0%)	33/187 (17.6%)	0.69
Balloon expandable	168/337 (49.9%)	79/150 (52.7%)	89/187 (47.6%)	0.36
SAPIEN	29/337 (8.6%)	13/150 (8.7%)	16/187 (8.6%)	0.97
SAPIEN 3	122/337 (36.2%)	58/150 (38.7%)	64/187 (34.2%)	0.40
SAPIEN XT	17/337 (5.0%)	8/150 (5.3%)	9/187 (4.8%)	0.83
Elective	302/336 (89.9%)	131/150 (87.3%)	171/186 (91.9%)	0.16
Transfemoral access	301/335 (89.9%)	133/149 (89.3%)	168/186 (90.3%)	0.75
Moderate sedation	208/336 (61.9%)	88/150 (58.7%)	120/186 (64.5%)	0.27
Discharge medication: antiplatelet agent (aspirin or P2Y12)	305/337 (90.5%)	132/150 (88.0%)	173/187 (92.5%)	0.16
Discharge medication: antithrombic agent (dabigatran, warfarin, factor Xa inhibitor)	100/337 (29.7%)	43/150 (28.7%)	57/187 (30.5%)	0.72
Device success^*∗*^	329/336 (97.9%)	147/149 (98.7%)	182/187 (97.3%)	0.47
Implantation success^†^	328/334 (98.2%)	145/150 (96.7%)	183/184 (99.5%)	0.09

Values are *n/N* (%) or mean ± standard deviation. NYHA = New York Heart Association; PROM = predicted risk of mortality; STS = Society of Thoracic Surgeons. ^*∗*^Defined as successful vascular access, delivery, and deployment of a single device in the proper anatomic location, appropriate performance of the prosthetic heart valve (aortic valve area >1.2 cm^2^ and mean aortic valve gradient <20 mm Hg or peak velocity <3 m/s without moderate or severe prosthetic valve aortic regurgitation), and successful retrieval of the delivery system. ^†^Defined as correct positioning of a single device in the proper anatomical location.

**Table 2:** Adverse events and outcomes.

	Overall (*n* = 337)	Persistent (*n* = 150)	Nonpersistent (*n* = 187)	*p* values
*30-day MACE*	34 (10.1)	18 (12.0)	16 (8.6)	0.30
Death	10 (3.0)	6 (4.0)	4 (2.1)	0.35
Myocardial infarction	4 (1.2)	1 (0.7)	3 (1.6)	0.63
Stroke	7 (2.1)	3 (2.0)	4 (2.1)	1.00
Valve-related rehospitalization	11 (3.3)	6 (4.0)	5 (2.7)	0.55
Cardiac arrest	6 (1.8)	4 (2.7)	2 (1.1)	0.41

*30-day adverse events*				
Coronary compression	1 (0.3)	0 (0.0)	1 (0.5)	1.00
Cardiac arrest	6 (1.8)	4 (2.7)	2 (1.1)	0.41
Major vascular complication	5 (1.5)	2 (1.3)	3 (1.6)	1.00
New conduction/native pacer disturbance requiring PPM	71 (21.1)	61 (40.7)	10 (5.3)	<0.001

*1-year mortality*				
Cardiac-related mortality	21 (6.2)	14 (9.3)	7 (3.7)	0.035
All-cause mortality	40 (11.9)	24 (16.0)	16 (8.6)	0.036

Values are *n* (%). ICD = implantable cardioverter defibrillator; PPM = pacemaker.

**Table 3:** Relationship between persistent and nonpersistent groups and MACE and mortality.

	Univariable		
	Hazard ratios	95% confidence interval	*p* values
30-day MACE	1.40	0.72–2.75	0.33
30-day mortality	1.88	0.53–6.65	0.33
1-year cardiac-related mortality	2.54	1.02–6.29	0.044
1-year all-cause mortality	1.90	1.01–3.59	0.046

^*∗*^MACE is defined as a composite of death, myocardial infarction (MI), stroke, valve-related hospitalization, and cardiac arrest.

## Data Availability

The participants of this study did not give written consent for their data to be shared publicly, so due to the sensitive nature of the research, supporting data are not available.

## Abbreviations

AVB: Atrioventricular block
ECG: Electrocardiogram
ICD: Implantable cardioverter defibrillator
LBBB: Left bundle branch block
LVEF: Left ventricular ejection fraction
PPM: Permanent pacemaker
TAVR: Transcatheter aortic valve replacement
EMR: Electronic medical record
LVOT: Left ventricular outflow tract.

## Supplementary Materials

Supplementary Materials Supplemental Table 1: new-onset conduction disturbances within 7 days after TAVR. A breakdown of conduction disturbances found within 7 days after TAVR implantation by the valve type. The *n* of 733 is the overall patient population, and the percentages are percentages of patients with a disturbance from this population. Supplemental Table 2: new-onset conduction disturbances within 7 Days after TAVR by the valve type. A breakdown of the most common new-onset conduction disturbances found within 7 days after TAVR implantation, by self-expanding vs. balloon-expandable valves. The *n* count for each subgroup is the overall patient population receiving the valve type, and the percentages are percentages of patients with a disturbance from this population.

## References

[1] Mack M. J., Leon M. B., Thourani V. H. (2019). Transcatheter aortic-valve replacement with a balloon-expandable valve in low-risk patients. *New England Journal of Medicine*.

[2] Popma J. J., Deeb G. M., Yakubov S. J. (2019). Transcatheter aortic-valve replacement with a self-expanding valve in low-risk patients. *New England Journal of Medicine*.

[3] Tsoi M., Tandon K., Zimetbaum P. J., Frishman W. H. (2021). Conduction disturbances and permanent pacemaker implantation after transcatheter aortic valve replacement: predictors and prevention. *Cardiology in Review*.

[4] Jørgensen T. H., De Backer O., Gerds T. A., Bieliauskas G., Svendsen J. H., Søndergaard L. (2019). Mortality and heart failure hospitalization in patients with conduction abnormalities after transcatheter aortic valve replacement. *Journal of the American College of Cardiology: Cardiovascular Interventions*.

[5] Husser O., Pellegrini C., Kessler T. (2016). Predictors of permanent pacemaker implantations and new-onset conduction abnormalities with the SAPIEN 3 balloon-expandable transcatheter heart valve. *Journal of the American College of Cardiology: Cardiovascular Interventions*.

[6] De Torres-Alba F., Kaleschke G., Diller G. P. (2016). Changes in the pacemaker rate after transition from Edwards SAPIEN XT to SAPIEN 3 transcatheter aortic valve implantation: the critical role of valve implantation height. *JACC: Cardiovascular Interventions*.

[7] Godin M., Eltchaninoff H., Furuta A. (2010). Frequency of conduction disturbances after transcatheter implantation of an Edwards Sapien aortic valve prosthesis. *The American Journal of Cardiology*.

[8] Poels T. T., Houthuizen P., Van Garsse L. A., Maessen J. G., de Jaegere P., Prinzen F. W. (2014). Transcatheter aortic valve implantation-induced left bundle branch block: causes and consequences. *Journal of Cardiovascular Translational Research*.

[9] Smith C. R., Leon M. B., Mack M. J. (2011). Transcatheter versus surgical aortic-valve replacement in high-risk patients. *New England Journal of Medicine*.

[10] Adams D. H., Popma J. J., Reardon M. J. (2014). Transcatheter aortic-valve replacement with a self-expanding prosthesis. *New England Journal of Medicine*.

[11] Reardon M. J., Van Mieghem N. M., Popma J. J. (2017). Surgical or transcatheter aortic-valve replacement in intermediate-risk patients. *New England Journal of Medicine*.

[12] Rodés-Cabau J., Ellenbogen K. A., Krahn A. D. (2019). Management of conduction disturbances associated with transcatheter aortic valve replacement: JACC scientific expert panel. *Journal of the American College of Cardiology*.

[13] Nazif T. M., Chen S., George I. (2019). New-onset left bundle branch block after transcatheter aortic valve replacement is associated with adverse long-term clinical outcomes in intermediate-risk patients: an analysis from the PARTNER II trial. *European Heart Journal*.

[14] Regueiro A., Abdul-Jawad Altisent O., Del Trigo M. (2016). Impact of new-onset left bundle branch block and periprocedural permanent pacemaker implantation on clinical outcomes in patients undergoing transcatheter aortic valve replacement: a systematic review and meta-analysis. *Circulation: Cardiovascular Interventions*.

[15] Mentias A., Saad M., Girotra S. (2019). Impact of pre-existing and new-onset atrial fibrillation on outcomes after transcatheter aortic valve replacement. *JACC: Cardiovascular Interventions*.

[16] Tracy C. M., Epstein A. E., Darbar D. (2013). 2012 ACCF/AHA/HRS focused update incorporated into the ACCF/AHA/HRS 2008 guidelines for device-based therapy of cardiac rhythm abnormalities: a report of the American College of Cardiology foundation/American heart association task force on practice guidelines and the heart Rhythm society. *Journal of the American College of Cardiology*.

[17] Mack M. J., Brennan J. M., Brindis R. (2013). Outcomes following transcatheter aortic valve replacement in the United States. *Journal of the American Medical Association*.

[18] Carroll J. D., Edwards F. H., Marinac-Dabic D. (2013). The STS-ACC transcatheter valve therapy national registry: a new partnership and infrastructure for the introduction and surveillance of medical devices and therapies. *Journal of the American College of Cardiology*.

[19] Kappetein A. P., Head S. J., Genereux P. (2012). Updated standardized endpoint definitions for transcatheter aortic valve implantation: the Valve Academic Research Consortium-2 consensus document. *European Heart Journal*.

[20] Sager S. J., Damluji A. A., Cohen J. A. (2016). Transient and persistent conduction abnormalities following transcatheter aortic valve replacement with the Edwards-Sapien prosthesis: a comparison between antegrade vs. retrograde approaches. *Journal of Interventional Cardiac Electrophysiology*.

[21] Alperi A., Rodés-Cabau J., Simonato M. (2021). Permanent pacemaker implantation following valve-in-valve transcatheter aortic valve replacement: VIVID registry. *Journal of the American College of Cardiology*.

[22] Sengupta A., Alexis S. L., Lee T. (2021). Cusp overlap technique: should it become the standard implantation technique for self-expanding valves?. *Current Cardiology Reports*.

[23] Barthélémy O., Redheuil A., Collet J. P. (2022). Cusp-overlapping projections in TAVR: where the left meets the right. *JACC: Cardiovascular Interventions*.

[24] Loewenstein I., Merdler I., Hochstadt A. (2022). Generational differences in outcomes of self-expanding valves for transcatheter aortic valve replacement. *Journal of Invasive Cardiology*.

[25] Kroon H. G., van Gils L., Ziviello F. (2022). Clinical consequences of consecutive self-expanding transcatheter heart valve iterations. *Netherlands Heart Journal*.

[26] Kaul S. (2020). Raising the evidentiary bar for guideline recommendations for TAVR: JACC review topic of the week. *Journal of the American College of Cardiology*.

[27] Toggweiler S., Kobza R. (2018). Pacemaker implantation after transcatheter aortic valve: why is this still happening?. *Journal of Thoracic Disease*.

