# Supplementary material for: Impact of New-Onset Conduction Disturbances following Transcatheter Aortic Valve Replacement on Outcomes: A Single-Center Study
Source: J Interv Cardiol. 2023 May 31;2023:5390338. doi: 10.1155/2023/5390338 (PMC10247319; doi:10.1155/2023/5390338)
Supplement: Supplementary Materials — Supplemental Table 1: new-onset conduction disturbances within 7 days after TAVR. A breakdown of conduction disturbances found within 7 days after TAVR implantation by the valve type. The n of 733 is the overall patient population, and the percentages are percentages of patients with a disturbance from this population. Supplemental Table 2: new-onset conduction disturbances within 7 Days after TAVR by the valve type. A breakdown of the most common new-onset conduction disturbances found within 7 days after TAVR implantation, by self-expanding vs. balloon-expandable valves. The n count for each subgroup is the overall patient population receiving the valve type, and the percentages are percentages of patients with a disturbance from this population. [file 5390338.f1.docx]

**Supplemental Table 1. New-Onset Conduction Disturbances Within 7 Days After TAVR**

|  | **LBBB** | **RBBB** | **IVCD** | **2:1 Block** | **3:1 Block** | **4:1 Block** | **Bifascicular Block** | **LAFB** | **LPFB** | **First-Degree AVB** | **Second-Degree AVB: Mobitz Type I** | **Second-Degree AVB: Mobitz Type II** | **Third-Degree AVB** | **Atrial Fibrillation/Flutter** |
| --- | --- | --- | --- | --- | --- | --- | --- | --- | --- | --- | --- | --- | --- | --- |
| Persistent | 74 (10%) | 7 (1%) | 55 (8%) | 0 | 0 | 0 | 4 (1%) | 8 (1%) | 0 | 38 (5%) | 0 | 0 | 80 (11%) | 9 (1%) |
| Non-Persistent | 96 (13%) | 17 (2%) | 51 (7%) | 4 (1%) | 0 | 0 | 12 (2%) | 32 (4%) | 6 (1%) | 60 (8%) | 3 (0%) | 0 | 8 (1%) | 37 (5%) |
| Total (n=733) | 170 (23%) | 24 (3%) | 106 (14%) | 4 (1%) | 0 | 0 | 16 (2%) | 40 (5%) | 6 (1%) | 98 (13%) | 3 (0%) | 0 | 88 (12%) | 46 (6%) |

AVB = atrioventricular block; IVCD = intraventricular conduction delay; LAFB = left anterior fascicular block; LBBB = left bundle branch block; LPFB = left posterior fascicular block; RBBB = right bundle branch block; TAVR = transcatheter aortic valve replacement

**Supplemental Table 2. New-Onset Conduction Disturbances Within 7 Days After TAVR by Valve Type**

|  | **LBBB** | **RBBB** | **IVCD** | **2:1 Block** | **3:1 Block** | **4:1 Block** | **Bifascicular Block** | **LAFB** | **LPFB** | **First-Degree AVB** | **Second-Degree AVB: Mobitz Type I** | **Second-Degree AVB: Mobitz Type II** | **Third-Degree AVB** | **Atrial Fibrillation/Flutter** |
| --- | --- | --- | --- | --- | --- | --- | --- | --- | --- | --- | --- | --- | --- | --- |
| Self-Expanding Valves (n=363) |  |  |  |  |  |  |  |  |  |  |  |  |  |  |
| Persistent | 39 (11%) | 5 (1%) | 28 (8%) | 0 | 0 | 0 | 4 (1%) | 5 (1%) | 0 | 20 (6%) | 0 | 0 | 36 (10%) | 3 (1%) |
| Non-Persistent | 49 (13%) | 7 (2%) | 19 (5%) | 2 (1%) | 0 | 0 | 4 (1%) | 14 (4%) | 5 (1%) | 44 (12%) | 2 (1%) | 0 | 8 (2%) | 18 (5%) |
| Balloon Expandable Valves (n=370) |  |  |  |  |  |  |  |  |  |  |  |  |  |  |
| Persistent | 35 (9%) | 2 (1%) | 27 (7%) | 0 | 0 | 0 | 0 (0%) | 3 (1%) | 0 | 18 (5%) | 0 | 0 | 44 (12%) | 6 (2%) |
| Non-Persistent | 47 (13%) | 10 (3%) | 32 (9%) | 2 (1%) | 0 | 0 | 8 (2%) | 18 (5%) | 1 (0%) | 16 (4%) | 1 (0%) | 0 | 0 | 19 (5%) |

AVB = atrioventricular block; IVCD = intraventricular conduction delay; LAFB = left anterior fascicular block; LBBB = left bundle branch block; LPFB = left posterior fascicular block; RBBB = right bundle branch block; TAVR = transcatheter aortic valve
